# The Design, Simulation and Fabrication of an Omnidirectional Inertial Switch with Rectangular Suspension Spring

**DOI:** 10.3390/mi12040440

**Published:** 2021-04-15

**Authors:** Wenguo Chen, Rui Wang, Huiying Wang, Shulei Sun

**Affiliations:** 1 The College of Information Engineering, Qujing Normal University, Qujing 655000, China; wangrui100865@163.com (R.W.); kingwhy2008@163.com (H.W.); 2 The College of Mechanical Engineering, Guizhou Institute of Technology, Guiyang 550003, China; bobsunstuy@gmail.com

**Keywords:** MEMS, inertial micro-switch, surface micromachining, contact time, rectangular suspension spring

## Abstract

An omnidirectional inertial switch with rectangular spring is proposed in this paper, and the prototype has been fabricated by surface micromachining technology. To evaluate the threshold consistency and stability of omnidirectional inertia switch, the stiffness of rectangular suspension springs is analyzed. The simulation result shows that the coupling stiffness of the rectangular spring suspension system in the non-sensitive direction is a little more than that in the sensitive direction, which indicated that the omnidirectional switching system’s stability is reinforced, attributed to the design of rectangular springs. The dynamic response simulation shows that the threshold of the omnidirectional inertial switch using the rectangular suspension spring has high consistency in the horizontal direction. The prototype of an inertial switch is fabricated and tested successfully. The testing results indicate even threshold distribution in the horizontal direction. The threshold acceleration of the designed inertial switch is about 58 g in the X direction and 37 g in the Z direction; the contact time is about 18 μs.

## 1. Introduction

The inertial switch is a passive device widely used for monitoring vibration and inertial impact in the field of internet of things (IoT), especially for the transport of special goods and drop detection, and the inertial switch shows excellent performance. For example, in reference [1], an acceleration-threshold switch for transportation applications was proposed by Sven Michaelis et al. in 2000. In 2005, Whitley M R et al. reported a shock sensor for health monitoring [2]. Luke J. Currano et al. proposed a latching ultra-low power shock sensor for acceleration monitoring in 2008 [3]. In 2009, Jui-Chang Kuo et al. proposed a passive inertial switch using multiwall carbon nanotube (MWCNT)–hydrogel composite integrated with an inductor/capacitor (L–C), and indicated that the inertial switches can be used for safety and protection in airbags, crash recorders, and arming and firing systems [4]. In 2017, Xu Q et al. reported an Inertial Microswitch under Reverse Directional Ultra-High g Acceleration for IoT Applications [5].

In the vibration monitoring system, uniaxial inertial devices are integrated into different directions to monitor the omnidirectional vibration. This traditional solution reduces the integration of the system and brings the disadvantages of centroid system errors and high costs. The omnidirectional inertial switch proposed in this paper provides a fully new solution for inertial collision system; the acceleration in all directions can be observed by one device.

Due to the advantages of an omnidirectional mechanical inertial switch in integrated system solutions, researchers have proposed various research schemes for the omnidirectional inertial switch from different perspectives. In 2007, Jean D J et al. applied for a patent for an omnidirectional switch with a latching structure [6]. A multidirectional-sensitive inertial switch was reported by Yang Z et al. in 2012 [7]. An omnidirectional inertial switch with a lant of mass is reported by Li Xiaojie et al. in 2012 [8]. A novel MEMS omnidirectional inertial switch with flexible electrodes was reported by Nie Weirong et al. in 2014 [9]. Du L et al. reported an inertial switch with a novel radial electrode for uniform omnidirectional sensitivity [10]. In the study of inertial switches, according to the classification of a movable electrode design, although the omnidirectional switch movable electrode had circular proof mass as sensitive components, the suspension mode of the proof mass is different: One is the movable electrode with a single ring spring extending from the center, the other is the movable electrode with multiple symmetrical springs hanging a circular proof mass. Based on the movable electrode design method, the corresponding fixed electrode can also be divided into two design methods: placing the fixed contact in the middle of the circular proof mass and distributing it around the proof mass. From analysis of the overall structure of the omnidirectional switch, the structure stability was found to be higher when the fixed electrode is placed in the center of the mass block and the suspension spring is placed around the mass block; otherwise, it can save the overall structure size. According to the contact mechanism classification of omnidirectional inertial switches, there are two types: latching and elastic contact. The latching structure outputs a continuous guide signal after a collision, while the elastic collision structure outputs a pulse signal. The latching structure is suitable for one-time application, while the elastic collision structure can be triggered repeatedly. In conclusion, it can be seen from the above research that, in addition to optimizing structural design, as an omnidirectional sensitive inertial device, how to achieve the threshold consistency of the device in the whole direction has always been a research hotspot for researchers, especially the threshold consistency of the device in the horizontal 360 degrees. To improve the consistency of the omnidirectional switch threshold, optimizing the movable electrode suspension system’s design and structural parameters is the most direct method. In the reported literature, researchers all choose S-spring as the movable electrode’s suspension structure, but S-spring has a large longitudinal and transverse stiffness ratio, which will lead to a sharp decrease in system stiffness at the position away from the longitudinal spring. The higher longitudinal and transverse stiffness ratio of S-spring increases the complexity of system design, when optimizing the omnidirectional inertial system’s consistency. Therefore, choosing a spring structure with lower longitudinal and transverse stiffness can simplify the structural design process and improve the omnidirectional system’s stability. In this paper, we present a new scheme to solve the threshold consistency of the omnidirectional inertial switch in the horizontal plan around 360 degrees.

In the omnidirectional inertial switch design, the threshold value of the inertial switch in the horizontal plane should be kept consistent to the maximum. In our previous work, the snake springs were used as a suspension structure for movable electrodes. The horizontal plane thresholds are basically the same by adjusting the structural parameters of the springs [11]. In this design, an inertial switch with rectangular suspension springs is further proposed. To analyze the device’s stability, the stiffness of the rectangular spring suspension system in all directions is analyzed by the simulation method. The simulation and test results show that the thresholds of the omnidirectional switch with rectangular springs are uniform in the horizontal direction.

## 2. Design and Simulation

### 2.1. Structural Design and Stiffness Simulation

**Figure 1:**
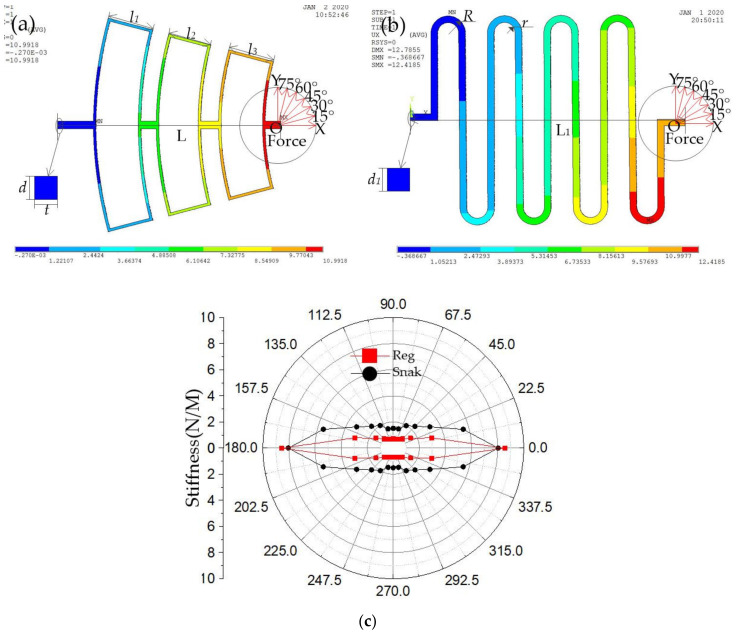
(**a**) Model of the rectangular spring; (**b**) Model of the S-shaped spring; (**c**) The stiffness distribution diagram of the rectangular spring and the S-shaped spring.

Before the device structure is designed, the rectangular spring and S-shaped spring is analyzed by the simulation method. The simulation conditions are set as follows: The nickel (Ni) is selected as a structure material, and Young’s modulus and density are chosen as 165 GPa and 8.96 g·cm^−3^ [12], respectively. The simulation models of springs are shown in Figure 1a,b. The main structural parameters of the simulation models are shown in Table 1. A force of 400 micro newtons is applied at the end of the spring in multiple directions, and the other end is fixed, the maximum deflection of the spring is obtained. The stiffness of designed springs is estimated by Formula (1) [13,14,15]:

(1) $$k=\frac{f}{x}\;N/M$$

where f is the constant force applied on the spring, x is the deformation due to the force, and k is the stiffness. The calculation results of the stiffness of rectangular spring and S-shaped spring around all directions is shown in Figure 1b. Simulation results shown in Figure 1c indicate that the stiffness of the rectangular spring is less than that of the S-shaped spring in the 22.5 degrees away from the x-axis when the rectangular spring has the same stiffness (≈9 N/M) as the S-shaped spring in the x-direction.

In conclusion, in the paper, the longitudinal stiffness is defined as the spring stiffness in the direction of 0 degree, and the transverse stiffness is defined as the spring stiffness in the direction of 90 degree. Figure 1c shows that the longitudinal and transverse stiffness ratio of the rectangular spring is greater than that of the S-shaped spring, which indicates that the rectangular spring suspension system is more stable when the suspension system is subjected to non-axial inertia impact.

**Figure 2:**
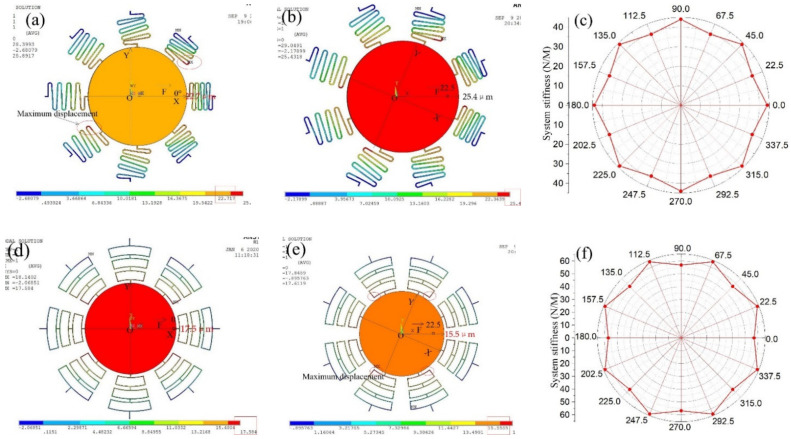
(**a**) Displacement distribution of suspension system when the force is applied in the x-direction. (**b**) Displacement distribution of suspension system with S-springs when the force is applied in the direction of 22.5 degrees. (**c**) The stiffness distribution of suspension system with S-shaped. (**d**) Displacement distribution of suspension system when the force is applied in the x-direction. (**e**) Displacement distribution of suspension system with rectangular springs when the force is applied in the direction of 22.5 degrees. (**f**) The stiffness distribution of suspension system with rectangular spring.

The stiffness of the suspension system with eight springs is simulated by a finite element model. A constant force of 1000 micronewtons is applied to the proof mass in the x-direction, the displacement of proof mass is about 22.7 μm, as shown in Figure 2a. The phenomenon that the position of maximum displacement of spring and proof mass is not coincident decreases the stability of elastic suspension system. The displacement is about 25.4 μm when the same force is applied to the proof mass in the direction of 22.5 degrees, as shown in Figure 2b. The stiffness distribution of suspension system with S-shaped is shown in Figure 2c, which indicates that the suspension system with S-shaped springs has maximum stiffness in the x-direction.

The displacement distribution of suspension system with rectangular springs when the force is applied in the x-direction is shown in Figure 2d, which indicated that the maximum displacement is 17.5 μm. The displacement distribution of the suspension system with rectangular springs when the force is applied in the direction of 22.5 degrees is shown in Figure 2e, and the displacement is about 15.5 μm. The stiffness distribution of the suspension system with rectangular springs is shown in Figure 2e, which indicates that the suspension system with rectangular springs has maximum stiffness in the direction of 22.5 degrees.

Through the stiffness comparison analysis of the system with the S-shaped springs and rectangular springs, the following conclusions can be drawn:

(1) The stiffness of the system with the S-shaped springs in the x-direction is greater than the direction of 22.5 degrees. To improve the threshold consistency of omnidirectional switches, the transverse stiffness of the S-springs can be improved by increasing the linewidth of springs [16]. However, the increasement of transverse stiffness of the S-spring also leads to the increasement of longitudinal stiffness of the S-spring. The system threshold consistency can also be improved by increasing the number of springs, which also increases the overall stiffness of the system.
(2) When the S-spring vibrates, the simulation results of maximum displacement show that the position coincidence degree between proof mass and springs is low, which decreases the stability of the system.
(3) The longitudinal and transverse stiffness ratio of the rectangular spring is greater than the S-shaped spring. The stiffness of the rectangular spring system in the direction of 22.5 degrees is slightly greater than that in the x direction, which indicates that the system has a strong ability to resist the inertial impact in the angle direction. Meanwhile, the maximum displacement of proof mass and springs has a high coincidence degree when the acceleration is applied to the system with rectangular springs.

Therefore, the comprehensive comparative analysis shows that the selection of rectangular spring is beneficial to improve the stability and threshold consistency of the omnidirectional inertial system. Meanwhile, based on the experimental results in this paper, it can be predicted that when the number of springs is reduced, the threshold consistency and stability of the system with rectangular springs will be higher than that of the system with the S-shaped springs.

### 2.2. Structure Design and Dynamic Response Simulation

**Figure 3:**
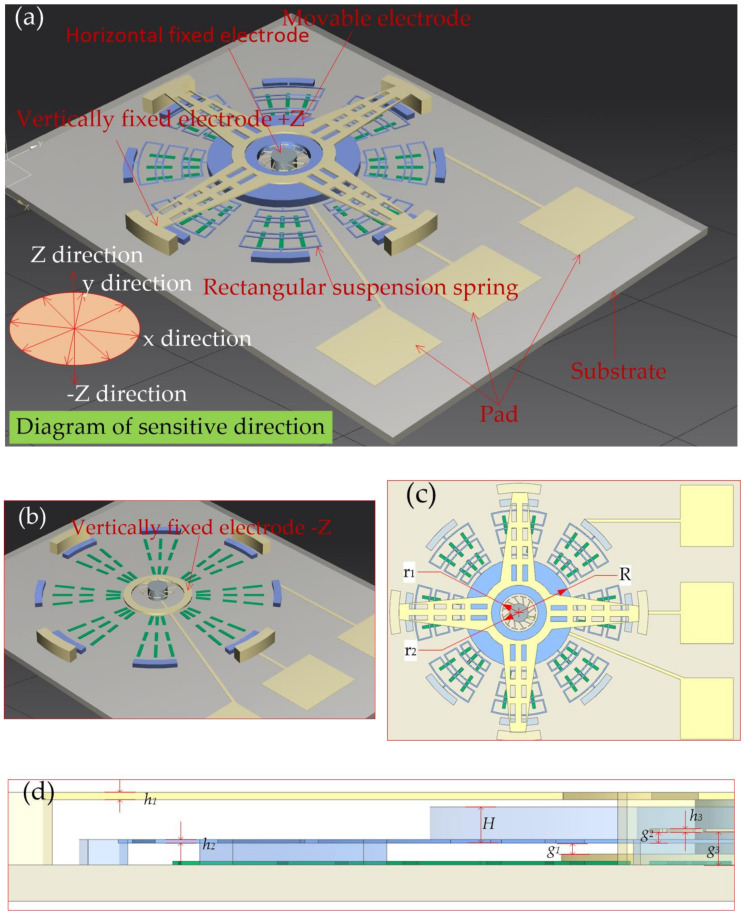
(**a**) The 3D structure of the designed inertial switch; (**b**) The schematic diagram of the fixed electrode; (**c**) The top view of the inertial switch structure; (**d**) The side view of a half structure.

According to the simulation analysis results of rectangular spring and S-spring stiffness, the rectangular spring is chosen as the elastic component in the designed inertial switch. The proof of the inertial switch is designed as ring-shaped. The 3D structure of the designed inertial switch is shown in Figure 3a. The circular mass block suspended by eight springs acts as a movable electrode. A dozen horizontal cantilevers fixed to a cylinder at the center of the block act as fixed electrodes for the inertial switch in the horizontal direction, as shown in Figure 3b. Vertically fixed electrodes are designed above and below the mass block, respectively, as shown in Figure 3b,c. When acceleration is applied to the inertial switch, the mass block moves towards the fixed electrode and comes in contact, causing the external circuit to switch. The proposed structural design in this paper can detect horizontal and vertical acceleration. The side view of a half structure is shown in Figure 3d. The main geometric parameters of the designed inertial micro-switch are shown in Table 2.

**Figure 4:**
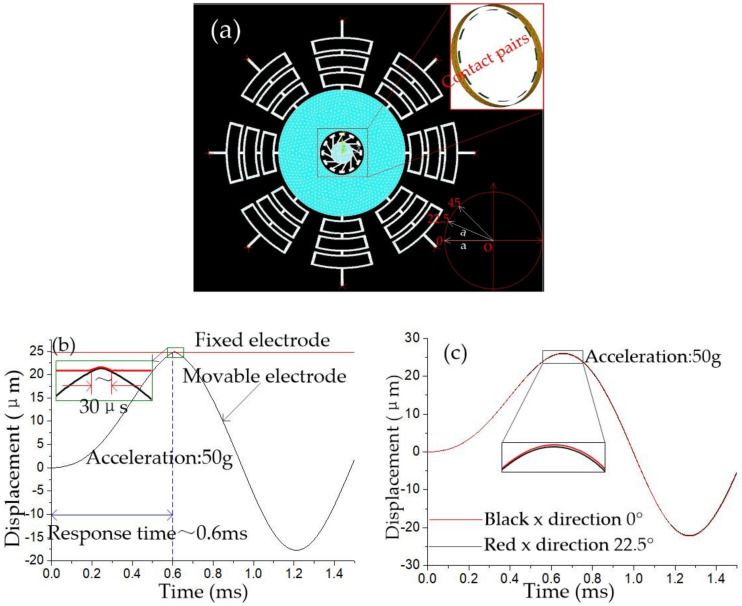
(**a**) FE model of the designed inertial switch; (**b**) The dynamic contact process in sensitive direction; (**c**) The dynamic contact process in the 0° and 22.5° directions.

The finite element model of an elastic collision system in the horizontal direction is shown in Figure 4a. The ends of eight rectangular springs are fixed, and the opposite surface of the fixed electrode and mass block is set as contact pairs. A series of half-sinusoidal accelerations with a pulse width of 1 millisecond and different peaks are applied to the inertial switch in the sensitive direction, and the dynamic contact process is shown in Figure 4b, which indicates that the threshold acceleration is about 50 g, and the response time and contact time is about 0.6 ms and 30 μs, respectively. Acceleration of 50 g is applied on the inertial switch in the 0° and 22.5° direction, respectively. The dynamic response process of the movable electrode is shown in Figure 4c. The dynamic curves of movable electrodes shown in Figure 4c indicate that movable electrodes’ vibration trajectories are kept in coincidence at 0° and 22.5° directions. This result demonstrated that the threshold of the designed inertial switch with eight rectangular springs has a high consistency value in all directions.

**Figure 5:**
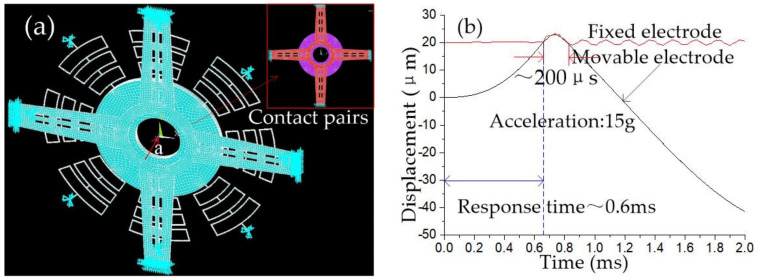
(**a**) The simulation finite element model for dynamic contact in the vertical direction; (**b**) The dynamic response curves of movable and fixed electrodes in the vertical direction.

The simulation finite element model for dynamic contact in the vertical direction is shown in Figure 5a. The cantilever beam’s lower surface and the mass block’s upper surface are set as contact pairs. The movable and fixed electrode’s dynamic response curves are shown in Figure 5b, which indicates that threshold acceleration is about 15 g in the vertical direction, and the contact time and response time is about 200 μs and 0.6 ms, respectively. The dynamic contact process shows that the movable electrode bounces in contact with the cantilever beam.

## 3. Fabrication and Test

**Figure 6:**
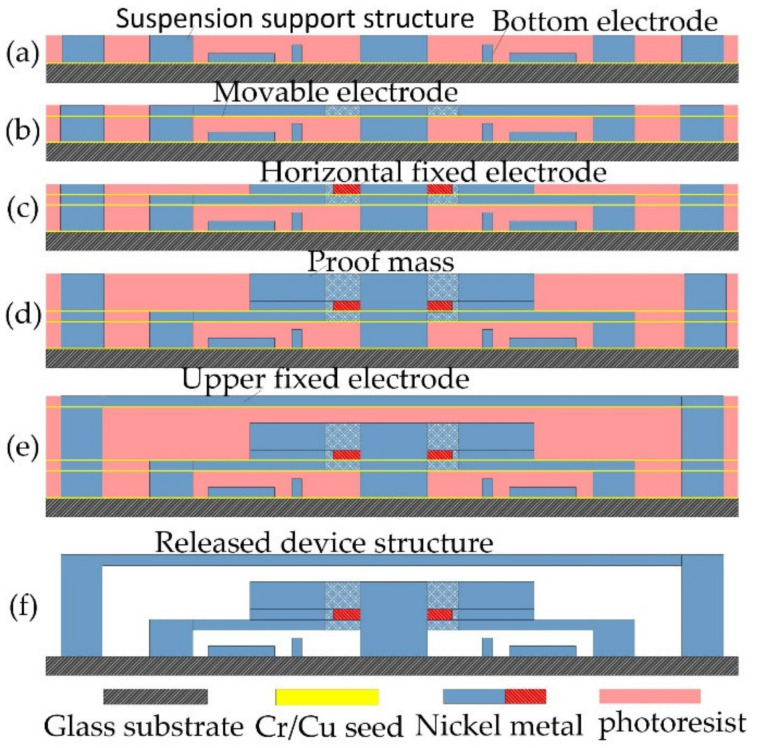
The diagram of fabrication.

The omnidirectional inertial switch is fabricated by surface micromachining technology with nickel metal. The main technology includes magnetron sputtering, etching, and electroplating, as shown in Figure 6.

(a) The Cr-Cu film was deposited on the glass substrate by magnetron sputtering technology, the bottom electrode was etched by spinning photoresist coating on the Cr-Cu thin film, and the suspended support structure of the movable electrode was prepared on the bottom electrode by repeated lithography technology. Finally, a metal structure is filled in the photoresist mould using an electroplating technique.
(b) The suspended spring and the underlying mass structure were prepared by repeated magnetron film sputtering, photoresist spin coating, lithography, and electroplating techniques.
(c) The third layer of the Cr-Cu film was fabricated and deposited and the horizontal fixed electrode was fabricated.
(d) The mass block is electroplated to a set thickness by repeated lithography and electroplating processes.
(e) The suspension interval is prepared above the mass block, and the upper fixed electrode is electroplated above the support structure.
(f) Finally, the photoresist was dissolved in sodium hydroxide liquid and the chrome-copper film was selectively removed to obtain the mechanical inertial switch structure.

**Figure 7:**
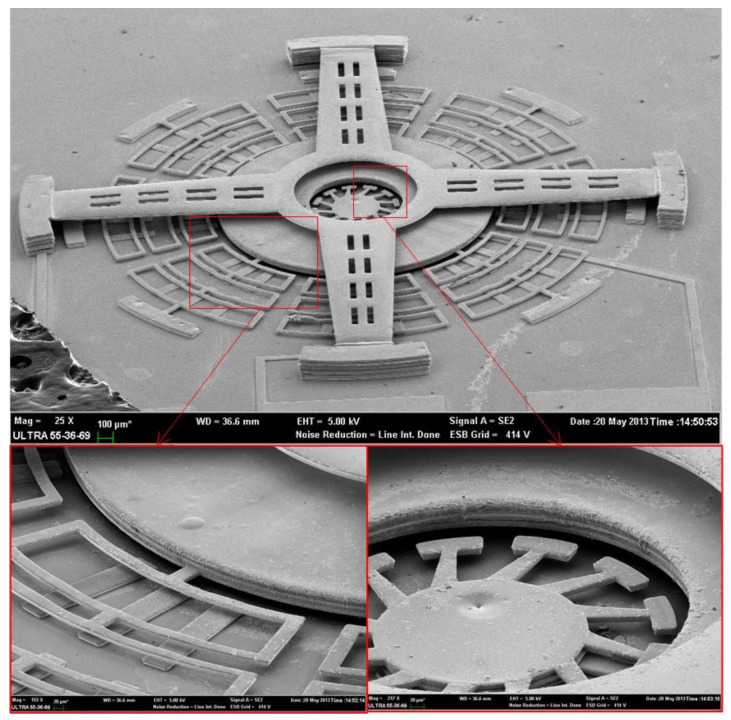
SEM photos of the prototype.

The fabricated prototype is fabricated successfully, as shown in Figure 7.

**Figure 8:**
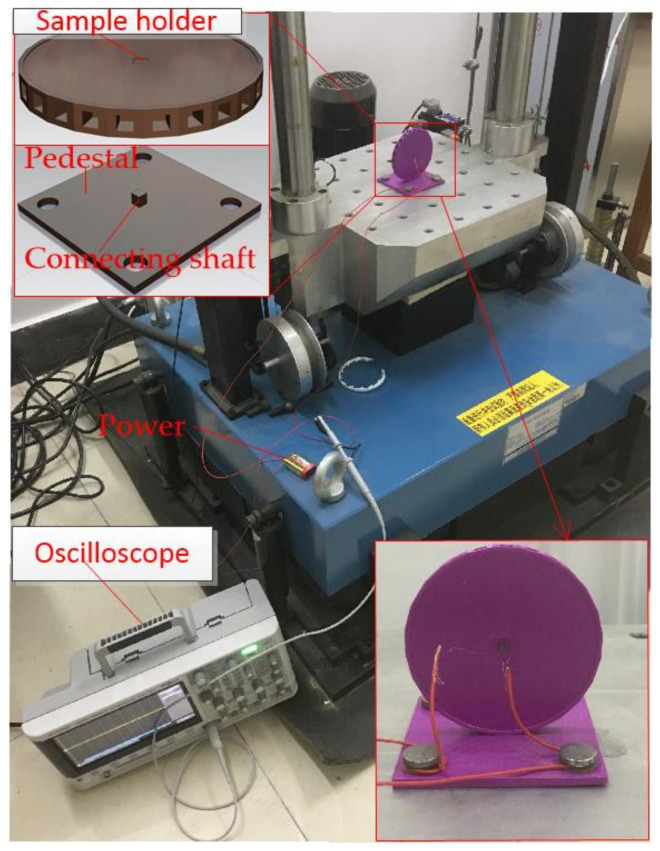
The dropping hammer system.

The prototype is packaged and tested by the dropping hammer system, as shown in Figure 8. The prototype is fixed on the fixture fabricated by 3D printing technology. The fixture is composed of two parts: a base plate fixed on a vibrating platform and a rotating circular objective table. The prototype is fixed on the objective table. The sensing direction of the device is perpendicular to the vibration plane. When the acceleration reaches the threshold value of the testing device, the inertial switch will be switched on trigger signal curves output to an oscilloscope.

**Figure 9:**
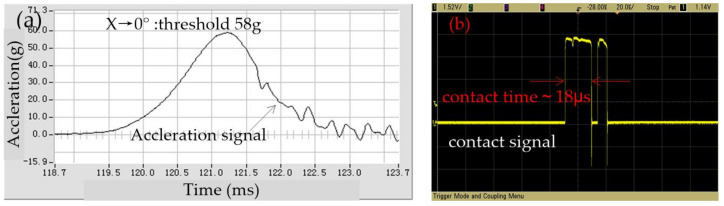
The tested threshold in the x direction.

**Figure 10:**
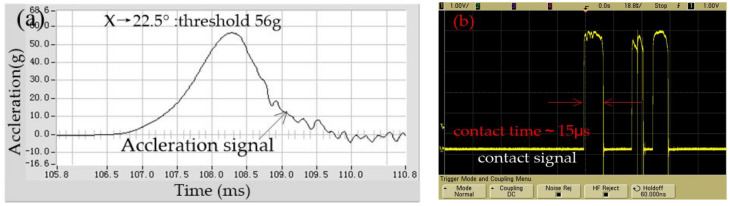
The tested threshold in 22.5 degrees away from the x-axis.

After repeated tests, it is shown that the output results of the tested samples are close to constant for several times; the representative test output data has been selected. The test result of the threshold in the x-direction is shown in Figure 9, which indicated that the threshold is about 58 g, while the first contact time is about 18 μs. The threshold is about 56 g in 22.5 degrees away from the X-axis, and the first contact time is about 15 μs, as shown in Figure 10. The comparative analysis of Figure 9 and Figure 10 shows that the dynamic contact signal of the prototype device bounces in the horizontal plane. Still, the threshold difference between the sensitive direction and the non-sensitive direction is about 2 g. This test results indicate that the fabricated omnidirectional inertial switch has a high threshold consistency in the horizontal omnidirectional range.

**Figure 11:**
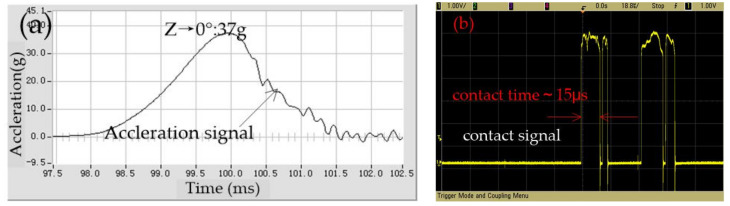
The tested threshold in the z direction.

The vertical direction threshold is tested, as shown in Figure 11, which indicated that the threshold is about 37 g in the z direction; the contact time is about 15 μs.

## 4. Conclusions

To improve the threshold consistency of the omnidirectional inertia switch in the horizontal direction, a suspension system design using rectangular spring as the elastic element is proposed in this paper. By comparing the stiffness of the S-shaped spring and the rectangular spring, it was seen that the latter had higher direction selectivity. The suspension system’s stiffness with rectangular and S-shaped springs is calculated by the simulation method, and the results indicated that the suspension system using rectangular springs has higher lateral impact resistance. Finally, a vibration impact platform was used to test the threshold values of the horizontal sensitive and non-sensitive directions of the prototype. The test results show that the threshold values of the prototype device in the horizontal plane are highly consistent, and the dynamic response process and threshold value are in accordance with the expected results of the dynamic simulation analysis. Compared with the results of the experiment and simulation analysis, the tested results show that bouncing signals appear in the contact signal because contact bounce mainly comes from the surface friction between the electrodes and the size parameter error in the preparation process. The threshold test results show that the test value is greater than the simulation value, especially the threshold value of the Z-direction, mainly because the pulse width of the acceleration applied in the test is about 3 ms, which is much larger than the simulation parameter value (1 ms).

## Figures and Tables

**Table 1:** The main geometric parameters of the designed springs.

Components	Rectangular Spring						S-Shaped Spring			
Geometric parameters	*L*	*l_1_*	*l_2_*	*l_3_*	*d*	*t*	*L_1_*	*R*	*r*	*d_1_*
Values (µm)	880	180	170	180	10	10	645	41	25	10

**Table 2:** The main geometric parameters of the designed inertial micro-switch.

Components	Proof Mass			Gap			Movable Electrode			Fixed Electrode		
Geometric parameters	*R*	*r_1_*	*H*	*g_1_*	*g_2_*	*g_3_*	*h_2_*	*b*	*t*	*h_1_*	*r_1_*	*h_3_*
Values (µm)	880	310	70	20	30	60	10	163	10	20	285	10
